# l-carnitine, a friend or foe for cardiovascular disease? A Mendelian randomization study

**DOI:** 10.1186/s12916-022-02477-z

**Published:** 2022-09-01

**Authors:** Jie V. Zhao, Stephen Burgess, Bohan Fan, C. Mary Schooling

**Affiliations:** 1 School of Public Health, Li Ka Shing Faculty of Medicine, The University of Hong Kong, 1/F, Patrick Manson Building, 7 Sassoon Road, Hong Kong SAR, China; 2 Medical Research Council Biostatistics Unit, University of Cambridge, Cambridge, UK; 3 Cardiovascular Epidemiology Unit, Department of Public Health and Primary Care, University of Cambridge, Cambridge, UK; 4 School of Public Health and Health Policy, City University of New York, New York, NY USA

**Keywords:** Carnitine, Mendelian randomization, Cardiovascular disease

## Abstract

**Background:**

l-carnitine is emerging as an item of interest for cardiovascular disease (CVD) prevention and treatment, but controversy exists. To examine the effectiveness and safety of l-carnitine, we assessed how genetically different levels of l-carnitine are associated with CVD risk and its risk factors. Given higher CVD incidence and l-carnitine in men, we also examined sex-specific associations.

**Methods:**

We used Mendelian randomization to obtain unconfounded estimates. Specifically, we used genetic variants to predict l-carnitine, and obtained their associations with coronary artery disease (CAD), ischemic stroke, heart failure, and atrial fibrillation, as well as CVD risk factors (type 2 diabetes, glucose, HbA1c, insulin, lipid profile, blood pressure and body mass index) in large consortia and established cohorts, as well as sex-specific association in the UK Biobank. We obtained the Wald estimates (genetic association with CVD and its risk factors divided by the genetic association with l-carnitine) and combined them using inverse variance weighting. In sensitivity analysis, we used different analysis methods robust to pleiotropy and replicated using an l-carnitine isoform, acetyl-carnitine.

**Results:**

Genetically predicted l-carnitine was nominally associated with higher risk of CAD overall (OR 1.07 per standard deviation (SD) increase in l-carnitine, 95% CI 1.02 to 1.11) and in men (OR 1.09, 95% CI 1.02 to 1.16) but had a null association in women (OR 1.00, 95% CI 0.92 to 1.09). These associations were also robust to different methods and evident for acetyl-carnitine.

**Conclusions:**

Our findings do not support a beneficial association of l-carnitine with CVD and its risk factors but suggest potential harm. l-carnitine may also exert a sex-specific role in CAD. Consideration of the possible sex disparity and exploration of the underlying pathways would be worthwhile.

**Supplementary Information:**

The online version contains supplementary material available at 10.1186/s12916-022-02477-z.

## Introduction

Cardiovascular disease (CVD) is a leading cause of death globally. Given the burden of CVD, identifying more effective intervention targets, especially sustainable dietary interventions applicable in daily life, is helpful for primary prevention and primary care. In the United States initial dietary recommendations designed in 1977 to reduce diet-related diseases, such as CVD, included reducing red meat consumption [1]. Red meat and the associated metabolites, such as trimethylamine-N-oxide (TMAO) [2], are gaining attention, not only for individual health but also for planetary health (https://eatforum.org/eat-lancet-commission/commissioners/). l-carnitine, the active form of dietary carnitine and a driver of TMAO, is emerging as a target for CVD prevention and treatment, because it has an important role in the oxidation of fatty acids and cardiac energy metabolism [3]. l-carnitine is abundant in animal products, especially red meat (https://ods.od.nih.gov/factsheets/Carnitine-HealthProfessional/#en1), and also promoted as a nutrient supplement to athletes for improving endurance performance, although such benefits have not been confirmed [4].

The effectiveness and safety of l-carnitine in CVD is unclear, with contradictory evidence showing potential cardiovascular benefits as well as raising safety concerns [3, 5]. Variants in l-carnitine related genes, *SLC22A4* and *SLC22A5*, are associated with ischemic heart disease [6]. People with variants in the *SLC22A5* gene (often referred to as carnitine transporter deficiency) which results in carnitine deficiency can have skeletal myopathy and/or cardiomyopathy [7]. l-carnitine is also relevant to testicular function [8, 9], when the role of testosterone in CVD is increasingly being investigated [10, 11], motivated by insights from evolutionary biology [12, 13], which would suggest sex-specific effects. In a systematic review and meta-analysis of 13 controlled trials in 3629 patients with acute myocardial infarction, l-carnitine was beneficial for angina but had no effect on the development of heart failure or myocardial infarction [14]. These findings might be partly explained by short follow-up, different doses of l-carnitine, and the inclusion of low-quality trials without randomization and blinding [14, 15]. Null effects for “hard outcomes” raise questions about the role of l-carnitine in CVD. In contrast, there are safety concerns that dietary carnitine may accelerate atherosclerosis via gut microbiota metabolites [16]. Specifically, in a prospective cohort study, l-carnitine was associated with higher risk of prevalent coronary artery disease, peripheral artery disease, and overall CVD, possibly mediated by its intestinal metabolite, TMAO [5]. However, observational studies are open to residual confounding, such as by socioeconomic position and health status, and cannot distinguish whether l-carnitine is a biomarker or a causal factor, making them difficult to use as a guide to interventions [17].

In this situation, using naturally occurring l-carnitine related genetic variants to predict serum carnitine in a Mendelian randomization (MR) study enables examination of the role of l-carnitine in an observational setting, without any potentially harmful interventions [17]. As genetic variants are determined at conception they are much less affected by the confounders that can bias conventional observational studies, such as socioeconomic position, so MR studies minimize confounding. MR is strongly recommended as a means of prioritizing drug targets for cardiovascular research, to avoid expensive failures in phase III randomized controlled trials (RCTs) [18, 19] and to identify mechanistic targets of intervention, complementary to RCTs [19]. Using MR, we examined the association of genetically predicted l-carnitine with CVD and its risk factors, and also conducted sex-specific analysis, where possible.

## Subjects and methods

### Study design

We conducted a two-sample MR study based on well-established large cohorts and consortia (Additional file 1: Table S1 and Additional file 2: Fig. S1). Specifically, we applied genetic proxies for l-carnitine to genome-wide association studies (GWAS) of CVD and its risk factors (type 2 diabetes, glucose, HbA1c, insulin, low-density lipoprotein (LDL)-cholesterol, high-density lipoprotein (HDL)-cholesterol, triglycerides, apolipoprotein B, and blood pressure). Given potential sex differences, we also conducted sex-specific analysis using individual data from the UK Biobank.

### Genetic predictors of l-carnitine

We identified independent (*r*^2^<0.01) genome-wide significant (5 × 10^−8^) single nucleotide polymorphisms (SNPs) from a recently published GWAS meta-analysis of l-carnitine in 23,658 people of European ancestry, with adjustment for age, sex, and study-specific covariates [20], where we identified 8 SNPs predicting l-carnitine (Additional file 1: Table S2). To check the validity of these selected SNPs as genetic instruments, we calculated the F-statistic, using a well-established formula [21]. A cut-off of 10 is usually used as a “rule of thumb” to distinguish between strong and weak instruments [22]; all the SNPs had F-statistics above 10. Detailed information about these SNPs is in Additional file 1: Table S2. To check whether these SNPs are associated with CVD or its risk factors via other phenotypes rather than via l-carnitine, we also assessed the association of these selected SNPs with potential causal factors for CVD, including Townsend index, education, smoking, and alcohol drinking in the UK Biobank, which are well-known factors affecting CVD and possibly l-carnitine. None of them were related to these factors in the UK Biobank at genome-wide significance (Additional file 1: Table S3). We also checked in Phenoscanner to examine if the genetic variant(s) were related to the outcomes directly, rather than via carnitine. rs1169299 and rs10466245 were also related to diabetes in DIAGRAM and HDL-cholesterol in GLGC respectively but did not reach genome-wide significance. rs1171617 was also related to uric acid. However, as suggested by a previous MR study [23], uric acid is downstream of l-carnitine, i.e., a mediator, so we did not exclude it as an invalid genetic instrument. In sensitivity analysis, we also included an isoform of l-carnitine, acetyl-carnitine, proxied by three genetic variants reaching genome-wide significance from the same GWAS as l-carnitine [20].

### Genetic associations with CVD and its risk factors

The data sources of all outcomes are shown in Additional file 1: Table S1.

#### Primary outcomes

The primary outcomes are as follows: fatal and non-fatal CVD events, including coronary artery disease (CAD), ischemic stroke, heart failure, and atrial fibrillation (AF).

Overall CAD was obtained from a large consortium, CARDIoGRAMplusC4D (42,096 cases, 99,121 controls) [24], as well as the UK Biobank (47,413 cases, 344,551 controls) and Finngen Biobank (21,012 cases, 197,780 controls). Ischemic stroke was obtained from MEGASTROKE consortium (34,217 cases and 406,111 controls) [25], the UK Biobank (7961 cases and 384,003 controls), and Finngen Biobank (10,551 cases and 208,241 controls) [26]. The associations of the genetic predictors for l-carnitine with CAD and ischemic stroke were obtained from UK Biobank using individual-level data. Heart failure was obtained from Heart Failure Molecular Epidemiology for Therapeutic Targets (HERMES) consortium, with 47,309 cases and 930,014 controls [27]. AF was obtained from the AF Consortium with 60,620 cases and 970,216 controls [28]. The latter two are meta-analyses of heart failure and AF which include the UK Biobank. The associations of the genetic predictors for l-carnitine with CAD and ischemic stroke were obtained from UK Biobank using individual-level data; genetic associations from other sources are from summary statistics. MR estimates from different data sources were meta-analyzed.

In sex-specific analysis of CVD events, we used individual-level data from UK Biobank. UK Biobank is a large, ongoing, prospective cohort study, with currently a median follow-up time of 11.1 years [29]. It recruited 502,713 people (intended to be aged 40–69 years, mean age 56.5 years, 45.6% men) from 2006 to 2010 in England, Scotland, and Wales, 94% of self-reported European ancestry. Incident CVD events were obtained from record linkage to hospitalization and death records; prevalent CVD events obtained from a nurse-led interview at recruitment were also included, as previously [11]. Genotyping was assessed using two similar arrays, i.e., the UK BiLEVE array and UK Biobank Axiom array. To control for population stratification, we only included participants with white British ancestry in the analysis. For quality control, we excluded participants who (1) have excess relatedness (more than 10 putative third-degree relatives), (2) have inconsistent information about sex based on genotyping and self-report, (3) have sex-chromosomes not XX or XY, (4) have poor-quality genotyping based on heterozygosity and missing rates, or (5) have withdrawn from UK Biobank. After quality control, we identified 47,413 cases of CAD (31,127 in men, 16,286 in women), 7961 cases of ischemic stroke (4915 in men, 3046 in women), 12,926 cases of heart failure (8456 in men, 4470 in women), and 18,382 cases of AF (12,260 in men, 6122 in women). Sex-specific associations with CAD, ischemic stroke, heart failure, and AF were obtained using logistic regression controlling for age, assay array, and 20 principal components.

#### Secondary outcomes

The secondary outcomes are as follows: type 2 diabetes, glucose, HbA1c, insulin, lipid profile (LDL-cholesterol, HDL-cholesterol, triglycerides and apolipoprotein B), systolic blood pressure, diastolic blood pressure, and body mass index (BMI), which are well-established risk factors for CVD in clinical practice, or recently identified to be related to CVD in MR, such as apolipoprotein B [30, 31].

In overall analysis, genetic associations with type 2 diabetes were obtained from DIAGRAM [32] (Additional file 1: Table S1). Genetic associations with glucose and HbA1c were obtained from UK Biobank and also a large consortium mainly in Europeans, MAGIC (140,595 people without diabetes for glucose [33] and ≤ 145,579 for HbA1c [34]). Genetic associations with insulin were obtained from MAGIC in 98,210 people [33]. Genetic associations with LDL-cholesterol, HDL-cholesterol, and triglycerides were obtained from UK Biobank and a large consortium mainly in Europeans, Global Lipids Genetics Consortium (GLGC) (188,577 participants of European descent and 7898 participants of non-European descent not taking lipid modulating medication [35]). Genetic associations with apolipoprotein B were obtained from UK Biobank. Genetic associations with BMI were obtained from the Genetic Investigation of ANthropometric Traits (GIANT) consortium in 681,275 participants of European ancestry [36]. In sex-specific analysis of CVD risk factors, genetic associations with diabetes were obtained from UK Biobank individual level data; genetic associations with insulin were obtained from the MAGIC; genetic associations with other risk factors were obtained from summary statistics in the Neale Lab GWAS of the UK Biobank, controlling for age, age^2^, and 20 principal components (Additional file 1: Table S1).

### Statistical analysis

We obtained the Wald estimate (genetic association with CVD and its risk factors divided by the genetic association with l-carnitine) for each SNP. We then combined SNP-specific estimates using inverse variance weighting with multiplicative random effects [37]. We aligned palindromic SNPs on allele and allele frequency and discarded any ambiguous SNPs (shown in Additional file 1: Table S2). We meta-analyzed the estimates from different data sources, where applicable. To account for multiple comparisons, we used a Bonferroni correction (corrected *p*-value: 0.05/15 = 0.003). Power calculations were conducted based on the approximation that the sample size for a MR study is the sample size for exposure on outcome divided by the *r*^2^ for genetic proxies on exposure [38, 39]. We repeated the analysis by sex and tested for differences between the estimates using a heterogeneity test [40].

To account for potential pleiotropy, in each analysis, we used different analytic methods robust to pleiotropy, including Mendelian randomization pleiotropy residual sum and outlier (MR-PRESSO), a weighted median, and weighted mode. MR-PRESSO applies a “leave-one-out” approach to identify the genetic variant(s) differentially driving the associations [41], i.e., outliers, and provide a corrected estimate after removing the outliers [42]. We present estimates from MR-PRESSO as the main results if there were outliers detected; otherwise, we used the IVW estimates. The weighted median provides a consistent estimate of the causal effect even when up to 50% of the information comes from genetic variants that are invalid instruments [43]. The weighted mode is based on the assumption that a plurality of genetic variants are valid instruments, i.e., no larger subset of invalid instruments estimating the same causal parameter than the subset of valid instruments exists [44]. All statistical analyses were conducted using the “TwoSampleMR” (for extracting genetic associations where publicly available and harmonization), “MendelianRandomization” and “MRPRESSO” (for deriving MR estimates), and “meta” package (for assessing heterogeneity in sex differences) in R (version 4.0.1, R Foundation for Statistical Computing, Vienna, Austria).

### Ethical approval

This research has been conducted using the UK Biobank Resource under application number 42468 and other large studies and consortia providing publicly available summary statistics (Additional file 1: Table S1 and Additional file 2: Fig. S1). The UK Biobank has already received the ethical approval from North West Multi-centre Research Ethics Committee (MREC) which covers the UK. It also got the approval from the Patient Information Advisory Group (PIAG) in England and Wales and from the Community Health Index Advisory Group (CHIAG) in Scotland. The study conforms to the ethical guidelines of the 1975 Declaration of Helsinki. The analysis of other publicly available summary statistics does not require additional ethical approval.

## Results

### Overall and sex-specific associations of genetically predicted l-carnitine with CVD

**Fig. 1:**
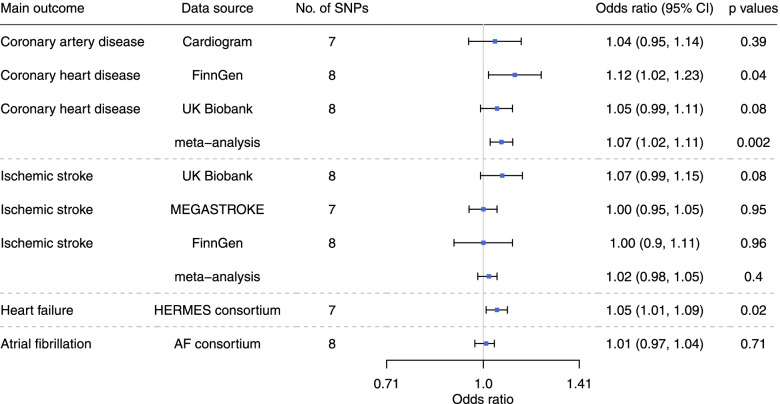
Genetically predicted l-carnitine (per standard deviation (SD) increase in l-carnitine) and cardiovascular disease (CVD) in the UK Biobank and large consortia

**Fig. 2:**
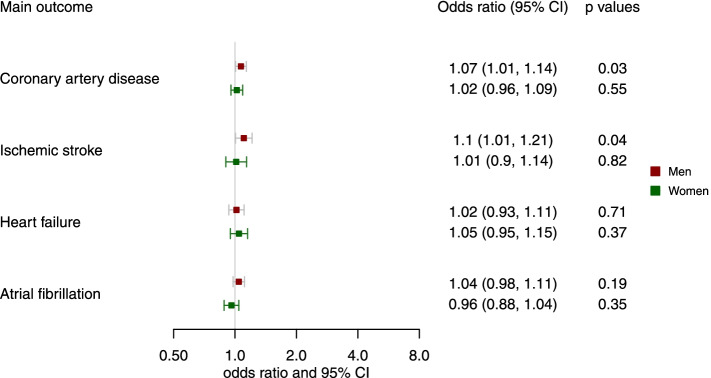
Genetically predicted l-carnitine (per SD increase in l-carnitine) and CVD by sex in the UK Biobank

Genetically predicted higher l-carnitine was nominally associated with higher risk of CAD and heart failure in men and women, with odds ratio (OR) 1.07 (95% confidence interval (CI) 1.02 to 1.11) for CAD and OR 1.05 (95% CI 1.01 to 1.09) for heart failure per standard deviation (SD) increase in genetically predicted l-carnitine (Fig. 1) but had null associations with ischemic stroke (OR 1.02, 95% CI 0.98 to 1.05) and AF (OR 1.01, 95% CI 0.97 to 1.04) (Fig. 1). As shown in Fig. 1, the association of genetically predicted l-carnitine with CAD was consistent in different data sources (CARDIoGRAMplusC4D, the UK Biobank and FinnGen). Genetically predicted l-carnitine was associated with higher risk of CAD in men (OR 1.07, 95% CI 1.01 to 1.14) but not women (OR 1.02, 95% CI 0.96 to 1.09), although the difference by sex did not reach statistical significance (*p* value for sex difference 0.31) (Fig. 2). There was heterogeneity for the IVW analysis of CAD for the overall analysis and for the UK Biobank in men (heterogeneity test *p* value < 0.05 as shown in Additional file 1: Table S4). MR-PRESSO detected outliers in the overall associations with CAD in FinnGen (shown in Additional file 1: Table S5), where we used corrected estimates from MR-PRESSO. The associations were robust to using the weighted median and weighted mode (Additional file 2: Fig. S2 and S3). Consistently, genetically predicted acetyl-carnitine was also related to higher risk of CAD (OR 1.06, 95% CI 1.01 to 1.10) and heart failure (OR 1.07, 95% CI 1.01 to 1.14) (Additional file 2: Fig. S4). In sex-specific analysis, as for l-carnitine, genetically predicted acetyl-carnitine was also related to higher risk of CAD in men (OR 1.09, 95% CI 1.02 to 1.16) but not in women (OR 1.00, 95% CI 0.92 to 1.09) (Additional file 2: Fig. S5).

### Overall and sex-specific associations of genetically predicted l-carnitine with CVD risk factors

**Fig. 3:**
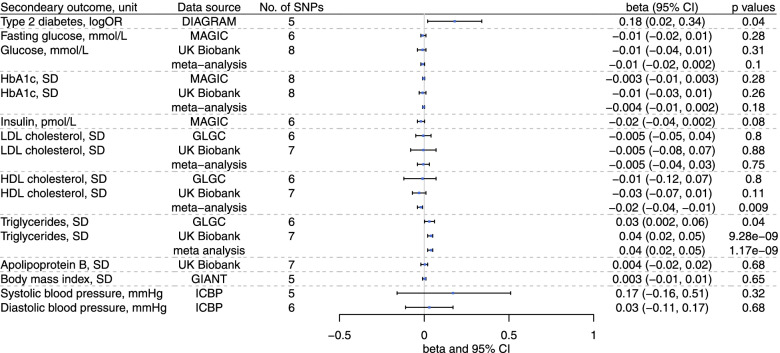
Genetically predicted l-carnitine (per SD increase in l-carnitine) and CVD risk factors in the UK Biobank and large consortia

Overall, genetically predicted higher l-carnitine was associated with higher triglycerides (effect size 0.04, 95% CI 0.02 to 0.05 per SD increase in genetically predicted l-carnitine) (Fig. 3), and nominally associated with lower HDL-cholesterol (− 0.02, 95% CI − 0.04 to − 0.01) (Fig. 3), Genetically predicted l-carnitine was nominally related to higher risk of diabetes (*p* = 0.04), but was not related to other glycemic traits (glucose, HbA1c or insulin). MR-PRESSO detected outliers in the overall associations with diabetes, LDL-cholesterol, HDL-cholesterol, apolipoprotein B and diastolic blood pressure, and sex-specific association with LDL-cholesterol, HDL-cholesterol, and apolipoprotein B (shown in Additional file 1: Table S5), where we used corrected estimates from MR-PRESSO. The associations were generally robust to using the weighted median and weighted mode (Additional file 2: Fig. S6). For genetically predicted acetyl-carnitine, we also found an association with higher triglycerides (0.06, 95% CI 0.04 to 0.07) and lower HDL-cholesterol (− 0.03, 95% CI − 0.05 to − 0.02) (Additional file 2: Fig. S7).

**Fig. 4:**
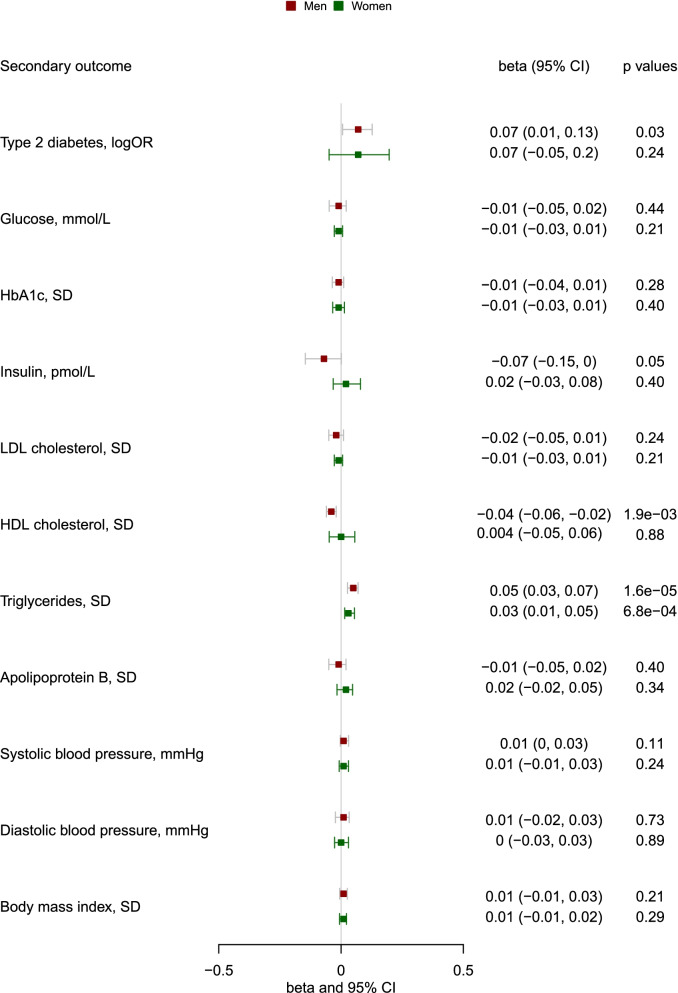
Genetically predicted l-carnitine (per SD increase in l-carnitine) and CVD risk factors by sex in the UK Biobank and MAGIC (for insulin)

Sex-specifically genetically predicted l-carnitine was related to higher triglycerides in men and women (Fig. 4), with no difference by sex (effect size 0.05, 95% CI 0.03 to 0.07 in men; effect size 0.03, 95% CI 0.01 to 0.05 in women). Genetically predicted higher l-carnitine was associated with lower HDL-cholesterol in men (effect size − 0.04, 95% CI − 0.06 to − 0.02), and not in women (0.004, 95% CI − 0.05 to 0.06, *p* value=0.88). Similar patterns of associations were evident using weighted median and weighted mode (Additional file 2: Fig. S8). Consistently, sex-specifically genetically predicted acetyl-carnitine was related to higher triglycerides in men and women (Additional file 2: Fig. S9), with no difference by sex. We also found genetically predicted higher acetyl-carnitine was associated with lower HDL-cholesterol in men and possibly also in women (Additional file 2: Fig. S9). The results of the power calculations are shown in Additional file 1: Table S6 [38, 39]. The detectable effect size is slightly smaller in men than in women.

## Discussion

Consistent with observational studies and systematic review and meta-analysis of RCTs [5, 14], our study suggests no benefit of l-carnitine for CVD or its risk factors. Instead, our findings suggest genetically predicted l-carnitine could be linked to higher risk of CAD overall and in men, and heart failure overall.

To our knowledge, this is the first MR to investigate the role of l-carnitine overall and sex-specifically. Using genetic proxies for l-carnitine minimizes confounding, which is challenging in observational studies. Our findings, together with observational evidence [5], do not support the use of l-carnitine in the primary prevention of CVD because of no benefit and the potential harm. We also found an association with CAD in men but not women. Consistently, we also found an association of genetically predicted l-carnitine and its isoform, acetyl-carnitine, with higher triglycerides and lower HDL-cholesterol. However, whether these factors are causally related to CAD has been questioned in MR studies [45, 46], and we found no difference by sex in the risk factors. As such, other mechanisms might exist. l-carnitine supplementation greatly increases TMAO [47], which has been considered on the biological pathway through which red meat intake increases cardiovascular risk [5]. The association of genetically predicted l-carnitine with CAD in men not in women is possibly due to a smaller number of CAD cases in women therefore a lower power (as shown in Additional file 1: Table S6). Meanwhile, the more obvious association in men has some consistency with higher CVD risk and higher carnitine in men than women [48]. The underlying mechanisms may involve factors with sex-specific roles in CVD, such as testosterone. Genetically predicted testosterone is associated with higher risk of CAD in men rather than in women [10]. Carnitine deficiency is associated with late-onset hypogonadism in men [49]; consistently, testosterone increases after l-carnitine supplementation [50].

Despite the novelty of this study, several limitations exist. First, this MR study examined the role of endogenous l-carnitine, which may not entirely correspond to exogenous carnitine supplementation*.* l-carnitine can be obtained from dietary sources such as beef, poultry, and dairy products, with the highest content of l-carnitine in red meat (2); l-carnitine increases after consuming food rich in carnitine, mainly red meat [5]. Second, some associations, including the association with CAD, did not reach Bonferroni-corrected significance, so we cannot exclude the possibility of a chance finding. However, the association was also shown for acetyl-carnitine, an isoform of l-carnitine, which adds confidence to the findings. Third, MR estimates, although less confounded, are less precise than conventional observational studies, because the genetic variants only explain a small proportion of the variance in exposure [38]. Replication in a larger sample, especially for the nominal associations, would be worthwhile. Fourth, MR requires stringent assumptions, i.e., the genetic variants are associated with the exposure, no confounders of the associations of the genetic variants with the outcomes exist, and the genetic variants are not associated with the outcomes other than via affecting the relevant exposure (no pleiotropy) [17]. To satisfy these assumptions, we only selected SNPs strongly associated with l-carnitine. The large heterogeneity for some outcomes, such as CAD, may suggest different mechanisms underlying the genetic associations or the existence of pleiotropy [37]. However, we used different analytic methods robust to pleiotropy, which gave a similar interpretation. Fifth, population stratification might affect MR estimates. However, the genetic associations with l-carnitine and with the outcomes are all from studies in people of European descent, with genomic control. Sixth, MR estimates reflect long-term effects, which may not be comparable with short-term effects of l-carnitine supplementation. Seventh, associations based on people of European ancestry might not apply to other populations, such as East Asians. However, causal effects are not expected to vary by setting [51], although the effect might be smaller in people with a lower intake of food rich in l-carnitine. The sex-specific associations were mostly based on the UK Biobank, which also need replication in other data sources when available. Eighth, the sex-specific genetic association with l-carnitine was not available; however, the genetics of most biomarker traits are shared between males and females [52]. Ninth, the role of l-carnitine might interact with microbiota [16]; however, information about microbiota is not available in the UK Biobank. Tenth, our study could be affected by survivor bias (selection bias). However, genetically predicted l-carnitine was not associated with longevity (data not shown). Nevertheless, different CVD subtypes share common risk factors, so it is possible that people dying of CAD were precluded from dying from stroke, heart failure or AF [53], which might underestimate the role of l-carnitine in these diseases. Finally, the relatively small effect size might not be of clinical significance. However, relatively small effects of causal factors may still be an important determinant of population health [54], particularly for foods often eaten daily.

From the perspective of clinical practice and dietary recommendation, our findings do not support a beneficial association of l-carnitine with CVD and its risk factors, but suggest potential harm. As such, dietary patterns lowering l-carnitine, such as lowering red meat intake, might be beneficial for CVD prevention. This study adds another piece of evidence to support lowering red meat consumption, which is also an environment-friendly lifestyle [55]. The more obvious association in men indicates more benefits from such dietary intervention might be achieved in men. Our study also raises safety concerns about carnitine supplements, especially in men, with direct relevance to those currently taking carnitine due to deficiency and for athletes and body-builders aiming to improve exercise performance. More studies assessing the safety and effectiveness of l-carnitine are needed.

## Conclusions

Our findings do not support a beneficial association of l-carnitine with CVD and its risk factors but suggest potential harm. l-carnitine may have a more obvious association with CAD in men. Consideration of the possible sex disparity and exploration of the underlying pathways would be worthwhile.

## Supplementary Information

**Additional file 1: Table S1.** Summary of genome-wide association studies included in this study. **Table S2.** Genetic predictors for l-carnitine and acetyl-carnitine. **Table S3.** Associations of genetic predictors for l-carnitine and acetyl-carnitine with potential confounders. **Table S4.** Heterogeneity statistics for overall and sex-specific analyses on genetically predicted l-carnitine and cardiovascular disease and its risk factors. **Table S5.** Outliers detected in MR-PRESSO for overall and sex-specific associations of genetically predicted l-carnitine with CVD and CVD risk factors. **Table S6.** Power calculation for the associations of genetically predicted l-carnitine and acetyl-carnitine with cardiovascular disease and its risk factors.

**Additional file 2: Fig. S1.** Flow chart of the data sources in the study. **Fig. S2.** Sensitivity analysis on genetically predicted l-carnitine and cardiovascular disease using different analytic methods. **Fig. S3.** Sensitivity analysis on genetically predicted l-carnitine and cardiovascular disease by sex using different analytic methods. **Fig. S4.** Genetically predicted acetyl-carnitine and cardiovascular disease overall. **Fig. S5.** Genetically predicted acetyl-carnitine and cardiovascular disease by sex. **Fig. S6.** Sensitivity analysis on genetically predicted l-carnitine and CVD risk factors overall using different analytic methods. **Fig. S7.** Genetically predicted acetyl-carnitine and cardiovascular disease overall. **Fig. S8.** Sensitivity analysis on genetically predicted l-carnitine and CVD risk factors by sex using different analytic methods. **Fig. S9.** Genetically predicted acetyl-carnitine and cardiovascular risk factors by sex.

## Data Availability

Data described in the manuscript will be available upon request and approval by the UK Biobank (https://www.ukbiobank.ac.uk/enable-your-research/apply-for-access).
