# Supplementary material for: l-carnitine, a friend or foe for cardiovascular disease? A Mendelian randomization study
Source: BMC Med. 2022 Sep 1;20:272. doi: 10.1186/s12916-022-02477-z (PMC9434903; doi:10.1186/s12916-022-02477-z)
Supplement: Supplementary file 1 — Additional file 1: Table S1. Summary of genome-wide association studies included in this study. Table S2. Genetic predictors for l-carnitine and acetyl-carnitine. Table S3. Associations of genetic predictors for l-carnitine and acetyl-carnitine with potential confounders. Table S4. Heterogeneity statistics for overall and sex-specific analyses on genetically predicted l-carnitine and cardiovascular disease and its risk factors. Table S5. Outliers detected in MR-PRESSO for overall and sex-specific associations of genetically predicted l-carnitine with CVD and CVD risk factors. Table S6. Power calculation for the associations of genetically predicted l-carnitine and acetyl-carnitine with cardiovascular disease and its risk factors. [file 12916_2022_2477_MOESM1_ESM.pdf]

Table S1. Summary of genome-wide association studies included in this study

Outcomes	Information of genome-wide association study (GWAS)		
Primary outcomes	Sources	Sample size	Reference and/or web link
Overall			
CAD	CARDIoGRAMplusC4D	42,096 cases, 99,121 controls	doi: 10.1038/ng.3396
	UK Biobank	47,413 cases, 344,551 controls	https://www.ukbiobank.ac.uk/
	FinnGen	21,012 cases, 197,780 controls	https://www.finngen.fi/fi
Ischemic stroke	MEGASTROKE consortium	34,217 cases and 406,111 controls	doi: 10.1038/s41588-018-0058-3.
	UK Biobank	7,961 cases and 384,003 controls	https://www.ukbiobank.ac.uk/
	FinnGen	10,551 cases and 208,241 controls	https://www.finngen.fi/fi
Heart failure	Heart Failure Molecular Epidemiology for Therapeutic Targets (HERMES) consortium	47,309 cases and 930,014 controls	doi: 10.1038/s41467-019-13690-5.
Atrial fibrillation	AF Consortium	60,620 cases and 970,216 controls	doi: 10.1038/s41588-018-0171-3
Sex-specific			
CAD	UK Biobank	31,127 cases in men, 16,286 cases in women	https://www.ukbiobank.ac.uk/
Ischemic stroke		4,915 cases in men, 3,046 cases in women	
Heart failure		8,456 cases in men, 4,470 cases in women	
Atrial fibrillation		12,260 cases in men, 6,122 cases in women	
Secondary outcomes			
Overall			
Diabetes	DIAGRAM	62,892 cases and 596,424 controls	doi: 10.1038/s41467-018-04951-w
Fasting glucose	MAGIC	140,595 (67,506 men and 73,089 women)	https://magicinvestigators.org/downloads/
Glucose	UK Biobank (analysis from Neal Lab round 2)	361,194	http://www.nealelab.is/uk-biobank
HbA1c	UK Biobank (analysis from Neal Lab round 2)	361,194	http://www.nealelab.is/uk-biobank
	MAGIC	145,579	doi: 10.1038/s41588-021-00852-9

insulin	MAGIC	98,210	https://magicinvestigators.org/downloads/ doi: 10.1038/s41467-020-19366-9
LDL-c, HDL-c and triglycerides	GLGC and UK Biobank (summary data in the published study)	188,577 participants of European descent and 7,898 participants of non-European descent;<br>440,546 in UK Biobank	http://csg.sph.umich.edu/willer/public/lipids2013/<br>doi: 10.1038/ng.2797<br>doi: 10.1371/journal.pmed.1003062
Apolipoprotein B	UK Biobank	440,546 in UK Biobank	doi: 10.1371/journal.pmed.1003062
SBP and DBP	Systolic blood pressure (SBP) GWAS meta-analysis of International Consortium of Blood Pressure (ICBP) and UK Biobank	757,601	doi: 10.1038/s41588-018-0205-x
BMI	Genetic Investigation of ANthropometric Traits (GIANT) consortium	681,275	doi: 10.1093/ije/dyu005
Sex-specific			
Diabetes	UK Biobank individual level data	17,710 cases in men, 11,488 cases in women	https://www.ukbiobank.ac.uk/
Other CVD risk factors	UK Biobank (analysis from Neal Lab round 2)	167,020 men, 194,174 women	http://www.nealelab.is/uk-biobank
Insulin	MAGIC	47,806 in men and 50,404 in women	

Table S2. Genetic predictors for L-carnitine and acetyl-carnitine

SNP*	gene	chromosome	EA	OA	EAF	Beta	SE	P value
L-carnitine								
rs1169299	*HNF1A*	12	C	T	0.46	0.06	0.01	3.86E-10
rs1171617	*SLC16A9*	10	T	G	0.76	0.43	0.01	6.53E-372
rs274551	*SLC22A5*	5	C	T	0.83	0.18	0.01	1.88E-48
rs77010315	*SLC36A2*	5	C	A	0.99	0.58	0.04	1.61E-39
rs10466245	*MARCH8*	10	G	A	0.77	0.09	0.01	4.81E-17
rs853358	*CD83*	6	T	A	0.21	0.10	0.01	1.48E-20
rs12715455	*SFMBT1*	3	T	A	0.36	0.07	0.01	1.24E-13
rs111653425	*SLC47A1*	17	T	C	0.01	0.28	0.04	2.31E-10
Acetyl-carnitine								
rs1171617	*SLC16A9*	10	T	G	0.76	0.27	0.01	9.96E-139
rs272869	*SLC22A4*	5	A	G	0.37	0.12	0.01	4.89E-35
rs149235996	*SLC36A2*	5	A	G	0.99	0.39	0.05	1.96E-17

SNP, single nucleotide polymorphism; EA, effect allele; OA, other allele; EAF, effect allele frequency; SE, standard error. *rs111653425 was not available for the overall analysis on L-carnitine and CAD in Cardiogram and ischemic stroke in MEGASTROKE, heart failure, diabetes, glucose in MGIC, lipids in GLGC, insulin, body mass index, and blood pressure. rs77010315 was not available in the overall analysis on L-carnitine and diabetes, glucose in MGIC, insulin and body mass index, systolic blood pressure. rs853358 was not available for the overall analysis for lipids in UK Biobank, body mass index and blood pressure, and was not used because of palindromic in the overall analysis for lipids in GLGC. Rs1171617 not available for overall analysis on diabetes. rs149235996 was not available for the overall and sex-specific analyses on acetyl-carnitine and diabetes, insulin, overall analysis on lipids in GLGC, glucose in MAGIC, and body mass index

Table S3. Associations of genetic predictors for L-carnitine and acetyl-carnitine with potential confounders

Potential confounders	SNP	chr	EA	OA	Beta	SE	P value
Townsend deprivation index at recruitment	rs77010315	5	A	C	0.002	0.009	0.82
	rs853358	6	T	A	0.002	0.003	0.33
	rs1169299	12	C	T	0.002	0.002	0.28
	rs1171617	10	T	G	0.006	0.002	0.02
	rs272869	5	G	A	-0.0001	0.002	0.95
	rs149235996	5	G	A	0.0005	0.010	0.96
	rs111653425	17	T	C	-0.005	0.009	0.56
	rs10466245	10	A	G	0.003	0.002	0.21
	rs274551	5	C	T	-0.001	0.003	0.67
	rs12715455	3	T	A	0.002	0.002	0.49
Current tobacco smoking	rs853358	6	T	A	-0.0004	0.001	0.81
	rs272869	5	G	A	0.002	0.001	0.09
	rs1169299	12	C	T	0.001	0.001	0.66
	rs149235996	5	G	A	0.002	0.005	0.7
	rs1171617	10	T	G	0.001	0.001	0.45
	rs111653425	17	T	C	0.000	0.005	0.99
	rs77010315	5	A	C	0.001	0.005	0.85
	rs274551	5	C	T	0.001	0.002	0.5
	rs10466245	10	A	G	0.002	0.001	0.11
	rs12715455	3	T	A	0.001	0.001	0.43
Alcohol intake frequency	rs149235996	5	G	A	-0.005	0.014	0.7
	rs1169299	12	C	T	0.006	0.003	0.054
	rs853358	6	T	A	-0.002	0.004	0.69
	rs274551	5	C	T	-0.008	0.004	0.058
	rs272869	5	G	A	0.001	0.003	0.75
	rs77010315	5	A	C	-0.005	0.014	0.7
	rs1171617	10	T	G	-0.002	0.004	0.58
	rs10466245	10	A	G	0.002	0.004	0.49
	rs111653425	17	T	C	-0.024	0.014	0.08
	rs12715455	3	T	A	0.007	0.003	0.03
Age completed full time education	rs10466245	10	A	G	-0.002	0.002	0.53
	rs77010315	5	A	C	0.023	0.010	0.018
	rs853358	6	T	A	-0.002	0.003	0.34
	rs272869	5	G	A	-0.003	0.002	0.22
	rs149235996	5	G	A	0.025	0.010	0.011
	rs111653425	17	T	C	0.008	0.010	0.4
	rs1171617	10	T	G	-0.004	0.002	0.12
	rs274551	5	C	T	0.003	0.003	0.34
	rs1169299	12	C	T	-0.002	0.002	0.35
	rs12715455	3	T	A	-0.008	0.002	0.0002

Table S4. Heterogeneity statistics for overall and sex-specific analyses on genetically predicted L-carnitine and cardiovascular disease and its risk factors

Outcome, data source/sex	Cochran's Q statistic	Heterogeneity p value
Primary outcomes		
Coronary artery disease (CAD), overall, Cardiogram	24.0	5.3E-4
Coronary heart disease, overall, FinnGen	21.6	0.003
Coronary heart disease, overall, UK Biobank	19.8	0.01
CAD, men	15.1	0.03
CAD, women	10.9	0.15
Ischemic stroke, overall, MEGASTROKE	2.4	0.87
Ischaemic stroke, overall, FinnGen	12.5	0.09
Ischaemic stroke, overall, UK Biobank overall	3.0	0.88
Ischaemic stroke, men	1.0	0.99
Ischaemic stroke, women	5.4	0.61
Atrial fibrillation, AF Consortium	9.7	0.21
Atrial fibrillation, men	7.7	0.36
Atrial fibrillation, women	3.6	0.82
Heart failure, HERMES consortium	6.2	0.40
Heart failure, men	9.8	0.20
Heart failure, women	3.7	0.81
Secondary outcomes		
Type 2 diabetes overall	38.9	7.4E-8
Diabetes, men	15.1	0.03
Diabetes, women	27.5	2.7E-4
Fasting glucose, overall, MAGIC	7.8	0.10
Glucose, overall, UK Biobank	20.9	0.004
Glucose, men	16.5	0.02
Glucose, women	7.9	0.34
HbA1c, overall, MAGIC	25.8	5.4E-4
HbA1c, overall, UK Biobank	5.6	0.58
HbA1c, men	15.9	0.03
HbA1c, women	20.8	0.004
Insulin, overall	7.0	0.22
Insulin, men	5.9	0.12
Insulin, women	4.0	0.26
LDL cholesterol, overall, GLGC	42.6	4.4E-8
LDL cholesterol, overall, UK Biobank	67.1	1.6E-12
LDL cholesterol, men	31.7	4.6E-5
LDL cholesterol, women	7.9	0.34
HDL cholesterol, overall, GLGC	187.7	7.9E-38
HDL cholesterol, overall, UK Biobank	44.4	1.9E-8
HDL cholesterol, men	54.2	2.1E-9
HDL cholesterol, women	73.4	3.0E-13

TG, overall, GLGC	6.9	0.23
TG, overall, UK Biobank	12.1	0.06
TG, men	13.0	0.07
TG, women	13.7	0.06
ApoB, overall, UK Biobank	50.9	3.1E-9
ApoB, men	31.4	5.1E-5
ApoB, women	34.0	1.7E-5
BMI, overall, GIANT	6.9	0.14
BMI, men	6.1	0.53
BMI, women	5.0	0.66
SBP, overall, ICBP	20.7	3.7E-4
SBP, men	8.5	0.29
SBP, women	13.5	0.06
DBP, overall, ICBP	53.0	3.4E-10
DBP, men	21.8	0.003
DBP, women	25.9	5.4E-4

Table S5. Outliers detected in MR-PRESSO for overall and sex-specific associations of genetically predicted L-carnitine with CVD and CVD risk factors

Outcome	Sex	Data source	Outliers	*p* value from outlier test
CAD	Overall	FinnGen	rs1169299	p=0.008
			rs274551	p=0.048
T2D	Overall	Diagram	rs1169299 rs274551	p<0.005
LDL	Overall	GLGC	rs1169299	p<0.006
LDL	Overall	UKB	rs10466245 rs1169299 rs1171617	Both p<0.007
			rs274551	Both p=0.01
	Men	UKB	rs111653425	p<0.008
HDL	Overall	UKB	rs10466245 rs1169299 rs1171617 rs12715455	All p<0.007
	Men	UKB	rs10466245 rs1169299	Both p<0.008
	Women	UKB	rs10466245	P<0.008
			rs1171617 rs12715455	Both p=0.02
ApoB	Overall	UKB	rs1169299	P<0.007
			rs274551	P=0.007
ApoB	Men	UKB	rs111653425	p<0.008
DBP	Overall	GWAS of DBP	rs10466245 rs1169299 rs12715455	All p<0.006

Table S6. Power calculation for the associations of genetically predicted L-carnitine and acetyl-carnitine with cardiovascular disease and its risk factors

Outcomes	Effect size detected per SD increase in genetically predicted L-carnitine or acetyl-carnitine*		
	In men and women	In men	In women
Coronary artery disease	0.04	0.08	0.10
Ischemic stroke	0.06	0.18	0.22
Atrial fibrillation	0.05	0.11	0.16
Heart failure	0.06	0.14	0.18
Type 2 diabetes	0.05	0.22	0.28
Glucose	0.04	0.07	0.06
HbA1c	0.04	0.07	0.06
Fasting Insulin	0.09	0.13	0.13
LDL cholesterol	0.04	0.07	0.06
HDL cholesterol	0.04	0.07	0.06
Triglycerides	0.04	0.07	0.06
Apolipoprotein B	0.04	0.07	0.06
Body mass index	0.03	0.07	0.06
Systolic blood pressure	0.03	0.07	0.06
Diastolic blood pressure	0.03	0.07	0.06

OR, odds ratio; SD, standard deviation

For binary outcomes (CVD and diabetes), power calculation was based on the ratio of cases to the controls and the variance explained by the genetic proxies (r^2). For continuous outcomes, power calculation was based on the number of participants in the study and the variance explained by the genetic proxies. The calculation of r^2 was based on 2×effect allele frequency (EAF)× (1-EAF) ×beta^2/SD^2. Based on this calculation, the r^2 for L-carnitine and acetyl-carnitine are both 0.01.

*The effect size shown in this table was in the scale of log odds ratio of CVD and diabetes, and SD of CVD risk factors except for diabetes. The effect size means that at the current samples size we had 80% power to detect a change of risk (measured in log odds ratio) for CVD and diabetes, and a change (measured in standard deviation (SD)) for other CVD risk factors, per SD increase in genetically predicted L-carnitine or acetyl-carnitine.
