# Supplementary material for: l-carnitine, a friend or foe for cardiovascular disease? A Mendelian randomization study
Source: BMC Med. 2022 Sep 1;20:272. doi: 10.1186/s12916-022-02477-z (PMC9434903; doi:10.1186/s12916-022-02477-z)

Figure S1. Flow chart of the data sources in the study

Genetic association with exposure

Genome-wide association study of L-carnitine (n=23,658) → SNP-carnitine

Genetic association with outcomes

Overall:
CARDIoGRAMplusC4D, UK Biobank, and FinnGen (for CAD)
MEGASTROKE consortium, UK Biobank and FinnGen (for ischemic stroke)
HERMES Consortium (for heart failure)
AF Consortium (for AF)
Sex-specific:
UK Biobank (individual level data)
Details of these data sources were shown in Supplemental Table 1

→

SNP-CVD
- coronary artery disease (CAD)
- ischemic stroke
- heart failure
- atrial fibrillation (AF)

Overall:
DIAGRAM (for diabetes)
MAGIC and UK Biobank (for glucose, HbA1c)
MAGIC (for insulin)
GLGC and UK Biobank (for lipids)
Meta-analysis of ICBP and UK Biobank (for blood pressure)
GIANT (for BMI)
Sex-specific:
UK Biobank (summary statistics from Neale Lab)
MAGIC (for insulin)
Details of these data sources were shown in Supplemental Table 1

→

SNP-CVD risk factors
- type 2 diabetes
- Glucose, HbA1c, insulin
- lipid profile (LDL-cholesterol, HDL-cholesterol, triglycerides and apolipoprotein B)
- systolic blood pressure and diastolic blood pressure
- body mass index (BMI)

Figure S2. Sensitivity analysis on genetically predicted L-carnitine and cardiovascular disease using different analytic methods

Main outcome	Data source	No. of SNPs	Odds ratio (95% CI)	p values
Coronary artery disease	Cardiogram	7	1.03 (0.98, 1.09)	0.28
			1.03 (0.98, 1.09)	0.27
			1.04 (0.93, 1.17)	0.42
Coronary artery disease	FinnGen	8	1.1 (1.02, 1.19)	0.01
			1.11 (1.02, 1.2)	0.01
			1.12 (1.02, 1.23)	0.04
Coronary artery disease	UK Biobank	8	1.04 (1.01, 1.08)	0.02
			1.04 (1.01, 1.08)	0.02
			1.05 (0.98, 1.12)	0.12
Ischemic stroke	UK Biobank	8	1.05 (0.97, 1.14)	0.21
			1.06 (0.97, 1.15)	0.21
			1.07 (1.01, 1.13)	0.03
Ischemic stroke	MEGASTROKE	7	1.01 (0.96, 1.06)	0.82
			1.01 (0.95, 1.06)	0.81
			1 (0.96, 1.04)	0.93
Ischemic stroke	FinnGen	8	1.01 (0.93, 1.11)	0.76
			1.02 (0.93, 1.11)	0.67
			1 (0.89, 1.13)	0.96
Heart failure	HERMES consortium	7	1.05 (1, 1.09)	0.03
			1.05 (1, 1.09)	0.03
			1.05 (1, 1.1)	0.06
Atrial fibrillation	AF consortium	8	1 (0.97, 1.04)	0.82
			1 (0.97, 1.04)	0.94
			1.01 (0.96, 1.05)	0.72

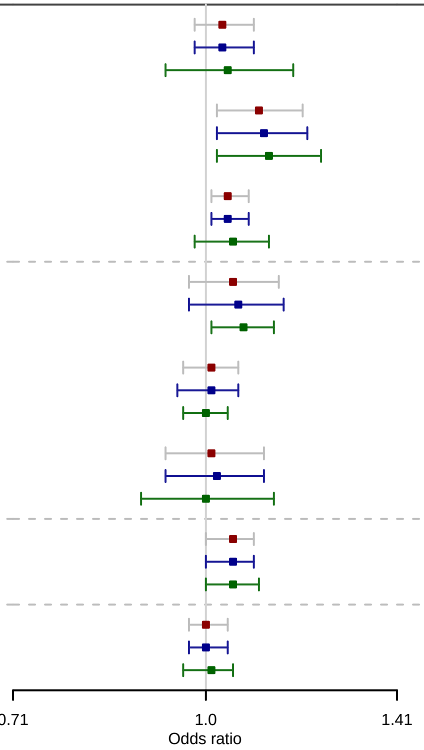

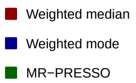

Figure S3. Sensitivity analysis on genetically predicted L-carnitine and cardiovascular disease by sex using different analytic methods

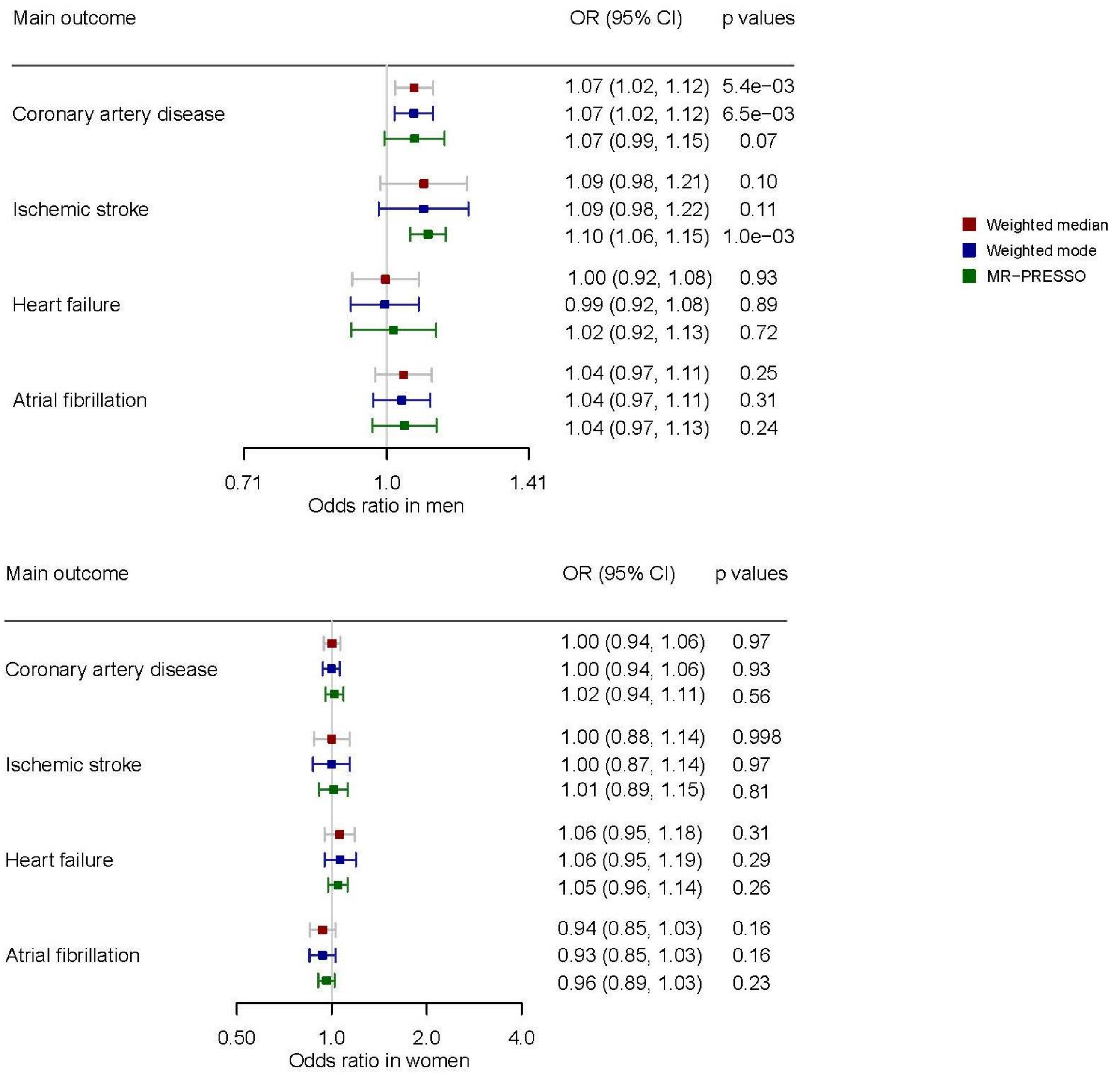

Figure S4. Genetically predicted acetyl-carnitine and cardiovascular disease overall

Main outcome	Data source	No. of SNPs	Odds ratio (95% CI)	p values
Coronary artery disease	Cardiogram	3	1.04 (0.96, 1.12)	0.34
Coronary heart disease	FinnGen	3	1.11 (1.00, 1.24)	0.06
Coronary heart disease	UK Biobank	3	1.05 (1.00, 1.11)	0.06
	meta−analysis		1.06 (1.01, 1.10)	0.01
Ischemic stroke	UK Biobank	3	1.05 (0.94, 1.19)	0.38
Ischemic stroke	MEGASTROKE	3	1.00 (0.93, 1.08)	0.97
Ischemic stroke	FinnGen	3	1.05 (0.92, 1.18)	0.48
	meta−analysis		1.02 (0.96, 1.08)	0.48
Heart failure	HERMES consortium	3	1.07 (1.01, 1.14)	0.02
Atrial fibrillation	AF consortium	3	1.01 (0.96, 1.06)	0.73

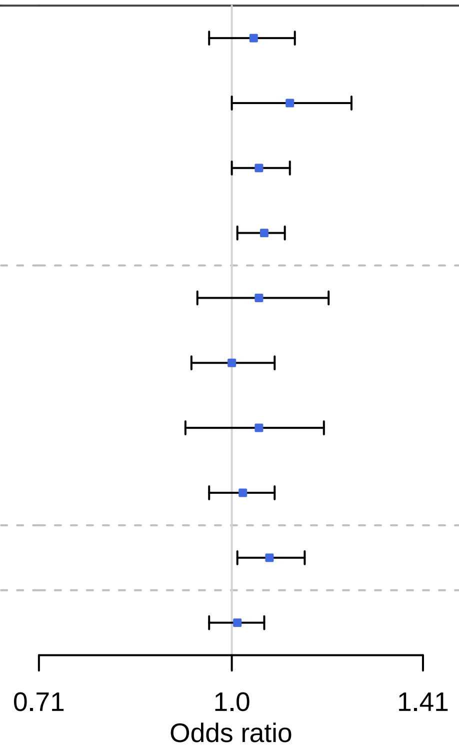

Figure S5. Genetically predicted acetyl-carnitine and cardiovascular disease by sex

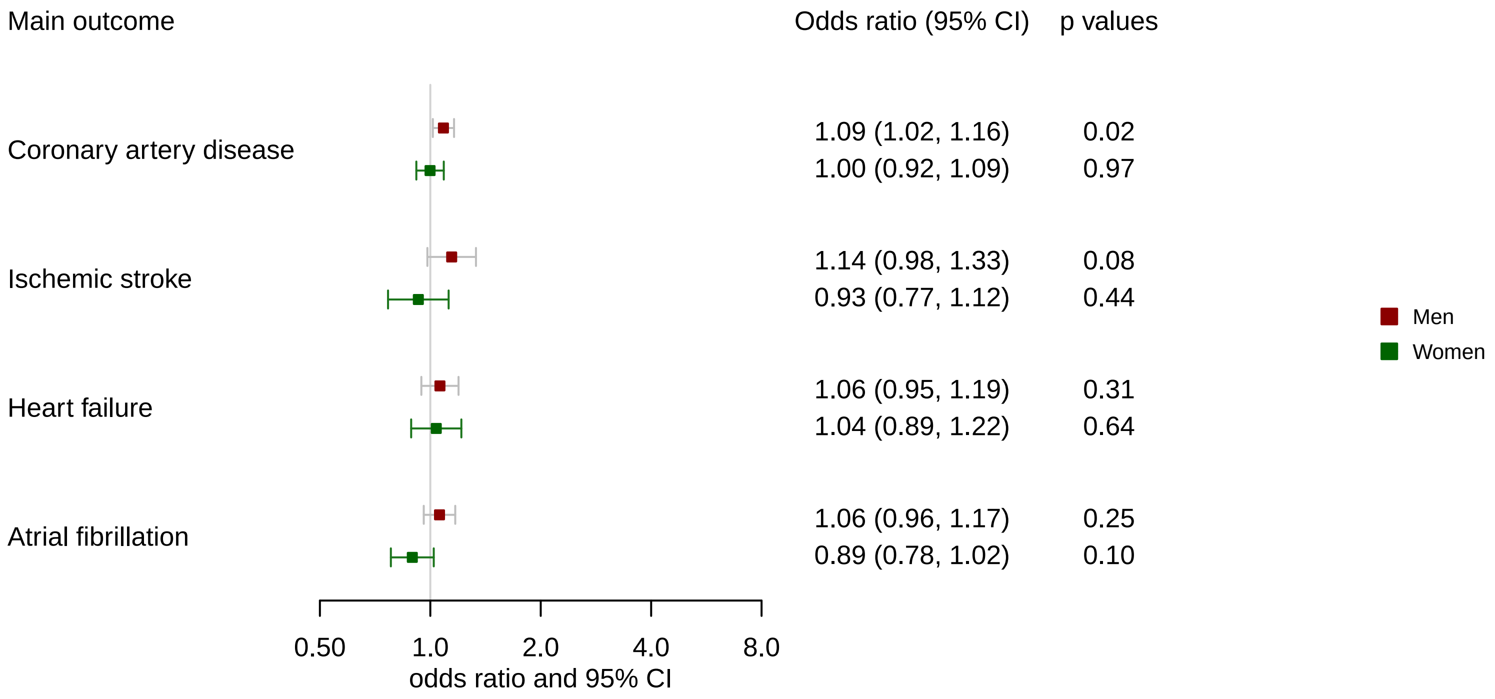

Figure S6. Sensitivity analysis on genetically predicted L-carnitine and CVD risk factors overall using different analytic methods

Weighted median Weighted mode MR−PRESSO

Secondeary outcome, unit	Data source	No. of SNPs	beta (95% CI)	p values
			0.12 (0.01, 0.23)	0.03
Type 2 diabetes, logOR	DIAGRAM	5	0.1 (−0.001, 0.2)	0.05
			0.18 (0.02, 0.34)	0.04
			−0.01 (−0.02, 0.005)	0.19
Fasting glucose, mmol/L	MAGIC	6	−0.01 (−0.02, 0.005)	0.19
			−0.01 (−0.03, 0.005)	0.31
			−0.02 (−0.04, −0.005)	0.01
Glucose, mmol/L	UK Biobank	8	−0.02 (−0.03, −0.003)	0.02
			−0.01 (−0.04, 0.02)	0.34
			−0.005 (−0.01, 0.002)	0.16
HbA1c, SD	MAGIC	8	−0.005 (−0.01, 0.002)	0.17
			−0.003 (−0.01, 0.003)	0.27
			−0.02 (−0.03, −0.005)	0.01
HbA1c, SD	UK Biobank	8	−0.02 (−0.03, −0.01)	0.004
			−0.01 (−0.04, 0.01)	0.3
			−0.02 (−0.03, 0)	0.06
Insulin, pmol/L	MAGIC	6	−0.02 (−0.03, 5e−04)	0.06
			−0.02 (−0.04, 0.01)	0.14
			0.01 (−0.02, 0.03)	0.72
LDL cholesterol, SD	GLGC	6	0.004 (−0.02, 0.03)	0.76
			−0.01 (−0.05, 0.04)	0.8
			0.003 (−0.01, 0.01)	0.56
LDL cholesterol, SD	UK Biobank	7	−0.001 (−0.01, 0.01)	0.92
			−0.005 (−0.08, 0.07)	0.88
			−0.02 (−0.04, 0.01)	0.23
HDL cholesterol, SD	GLGC	6	−0.01 (−0.04, 0.02)	0.56
			−0.03 (−0.12, 0.07)	0.53
			−0.02 (−0.03, −0.01)	1.68e−05
HDL cholesterol, SD	UK Biobank	7	−0.02 (−0.03, −0.01)	1.09e−05
			−0.03 (−0.07, 0.01)	0.11
			0.03 (0, 0.05)	0.05
Triglycerides, SD	GLGC	6	0.02 (−0.005, 0.05)	0.1
			0.03 (−0.01, 0.07)	0.09
			0.04 (0.03, 0.05)	1.42e−12
Triglycerides, SD	UK Biobank	7	0.04 (0.03, 0.05)	2.49e−12
			0.04 (0.02, 0.05)	0.001
			0.004 (−0.01, 0.01)	0.5
Apolipoprotein B	UK Biobank	7	0.001 (−0.01, 0.01)	0.9
			0.004 (−0.02, 0.02)	0.68
			0.004 (−0.01, 0.01)	0.43
Body mass index, SD	GIANT	5	0.003 (−0.01, 0.01)	0.45
			0.003 (−0.01, 0.02)	0.68
			0.15 (0, 0.31)	0.05
Systolic blood pressure, mmHg	ICBP	5	0.14 (−0.02, 0.3)	0.08
			0.17 (−0.31, 0.65)	0.37
			0.04 (−0.05, 0.13)	0.36
Diastolic blood pressure, mmHg	ICBP	6	0.02 (−0.07, 0.11)	0.65
			0.03 (−0.11, 0.17)	0.68

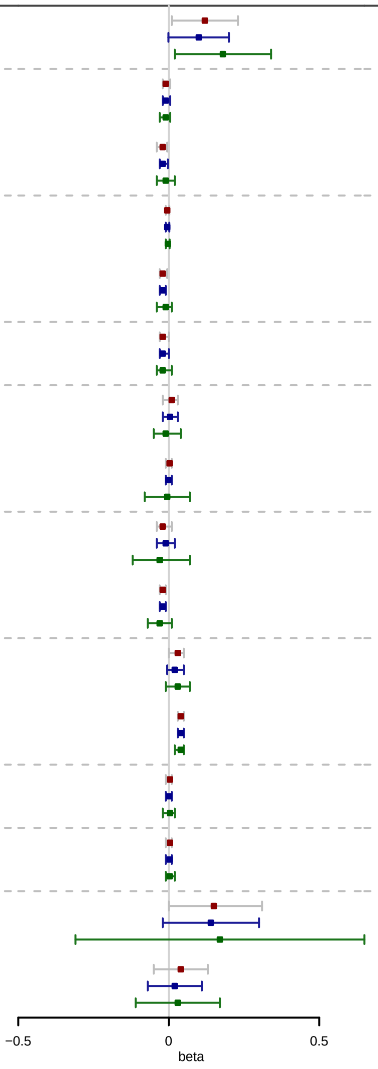

Figure S7. Genetically predicted acetyl-carnitine and cardiovascular disease overall

Secondeary outcome, unit	Data source	No. of SNPs	beta (95% CI)	p values
Type 2 diabetes, logOR	DIAGRAM	1	0.09 (−0.03, 0.22)	0.14
Fasting glucose, mmol/L	MAGIC	2	−0.02 (−0.04, 0.001)	0.06
Glucose, mmol/L	UK Biobank	3	−0.02 (−0.04, 0.001)	0.07
	meta−analysis		−0.02 (−0.03, −0.01)	0.01
HbA1c, SD	MAGIC	3	−0.001 (−0.01, 0.01)	0.83
HbA1c, SD	UK Biobank	3	−0.02 (−0.04, −0.01)	0.01
	meta−analysis		−0.01 (−0.03, 0.01)	0.32
Insulin, pmol/L	MAGIC	2	−0.01 (−0.04, 0.01)	0.26
LDL cholesterol, SD	GLGC	2	−0.003 (−0.04, 0.04)	0.88
LDL cholesterol, SD	UK Biobank	3	−0.004 (−0.02, 0.01)	0.56
	meta−analysis		−0.004 (−0.02, 0.01)	0.55
HDL cholesterol, SD	GLGC	2	−0.02 (−0.06, 0.01)	0.24
HDL cholesterol, SD	UK Biobank	3	−0.04 (−0.05, −0.02)	2.1e−07
	meta−analysis		−0.03 (−0.05, −0.02)	1.43e−07
Triglycerides, SD	GLGC	2	0.05 (0.01, 0.08)	0.005
Triglycerides, SD	UK Biobank	3	0.06 (0.04, 0.07)	2.83e−15
	meta analysis		0.06 (0.04, 0.07)	5.98e−17
Apolipoprotein B, SD	UK Biobank	3	−0.01 (−0.02, 0.01)	0.51
Body mass index, SD	GIANT	2	0.01 (0.001, 0.03)	0.03
Systolic blood pressure, mmHg	ICBP	3	0.26 (0.04, 0.49)	0.02
Diastolic blood pressure, mmHg	ICBP	3	0.23 (0.1, 0.35)	5e−04

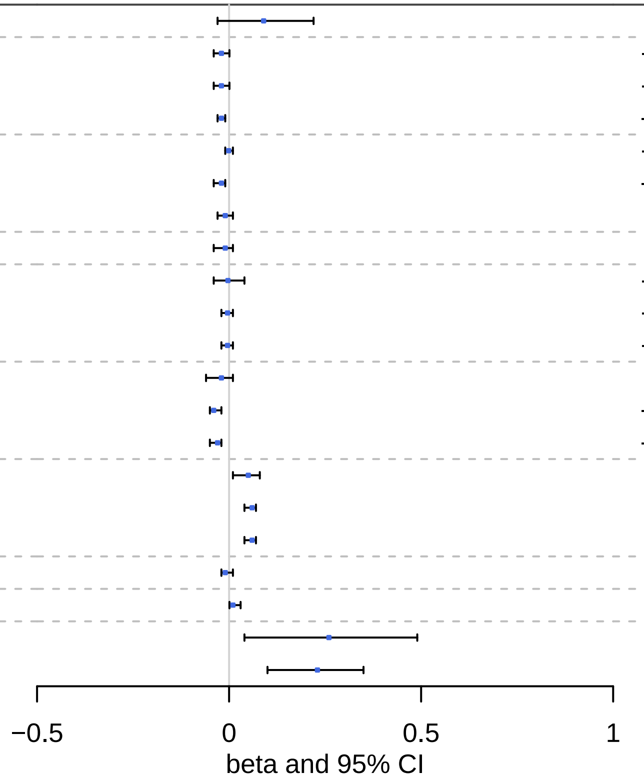

Figure S8. Sensitivity analysis on genetically predicted L-carnitine and CVD risk factors by sex using different analytic methods

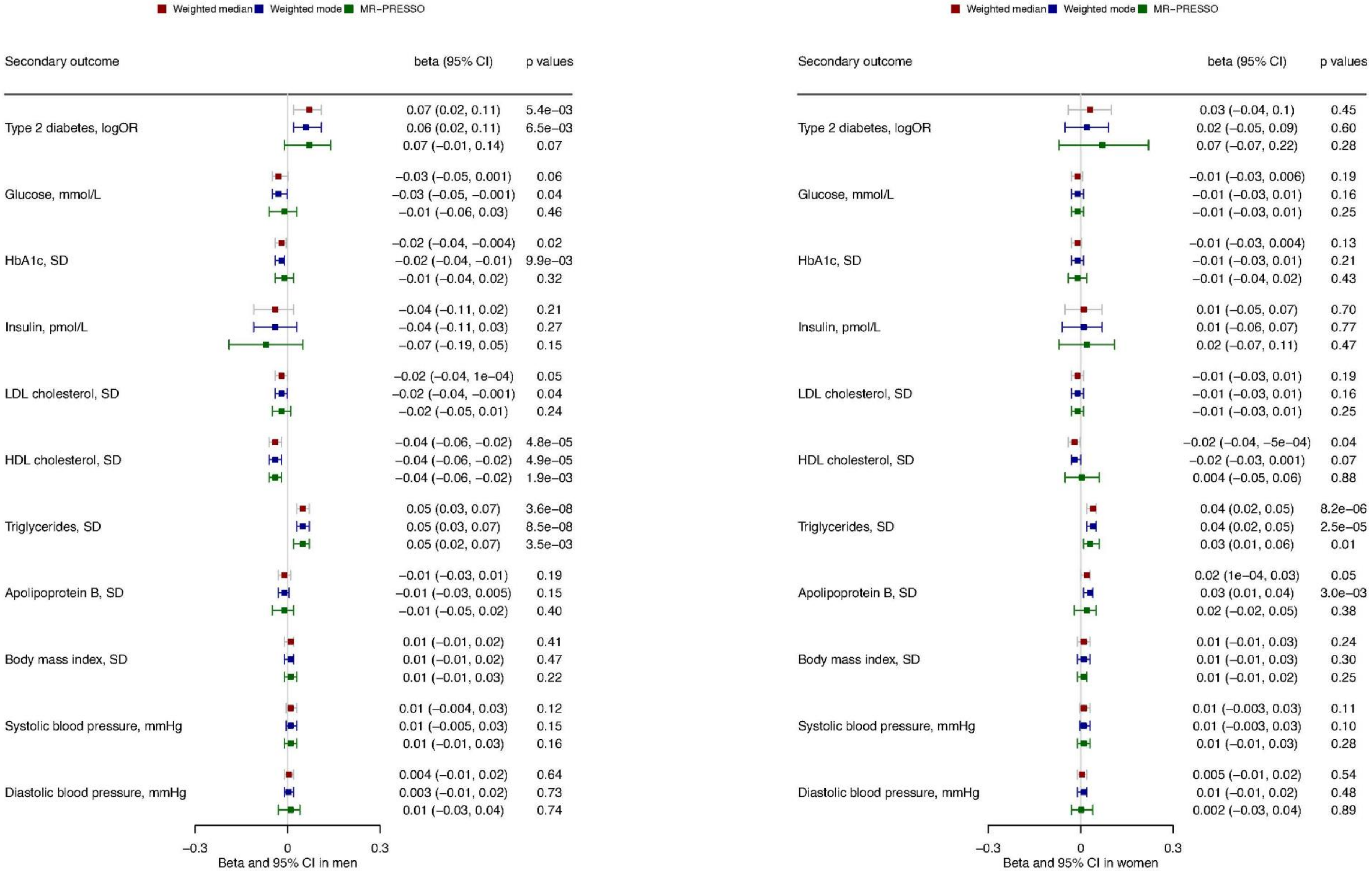

Figure S9. Genetically predicted acetyl-carnitine and cardiovascular risk factors by sex

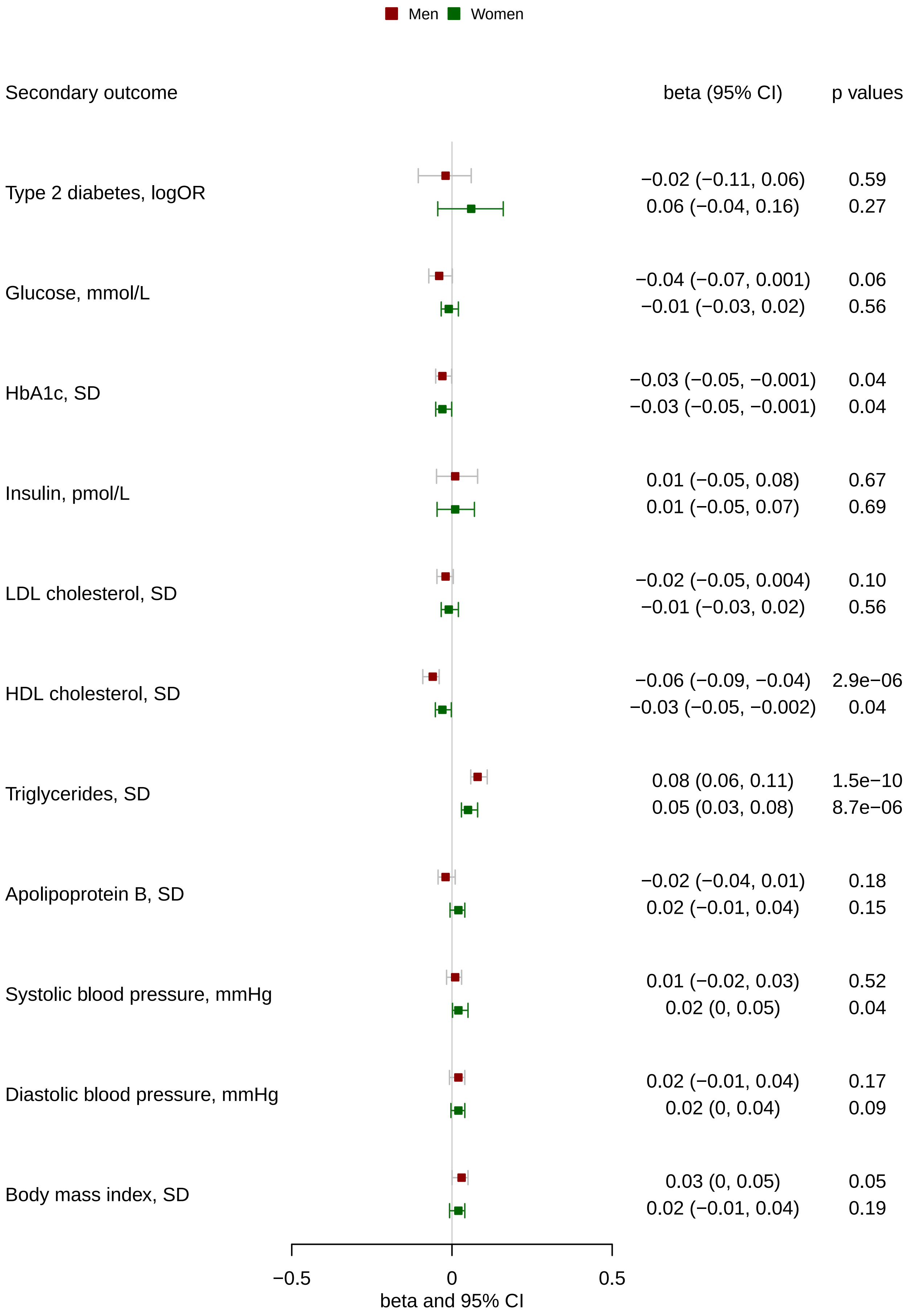

Supplement: Supplementary file 2 — Additional file 2: Fig. S1. Flow chart of the data sources in the study. Fig. S2. Sensitivity analysis on genetically predicted l-carnitine and cardiovascular disease using different analytic methods. Fig. S3. Sensitivity analysis on genetically predicted l-carnitine and cardiovascular disease by sex using different analytic methods. Fig. S4. Genetically predicted acetyl-carnitine and cardiovascular disease overall. Fig. S5. Genetically predicted acetyl-carnitine and cardiovascular disease by sex. Fig. S6. Sensitivity analysis on genetically predicted l-carnitine and CVD risk factors overall using different analytic methods. Fig. S7. Genetically predicted acetyl-carnitine and cardiovascular disease overall. Fig. S8. Sensitivity analysis on genetically predicted l-carnitine and CVD risk factors by sex using different analytic methods. Fig. S9. Genetically predicted acetyl-carnitine and cardiovascular risk factors by sex. [file 12916_2022_2477_MOESM2_ESM.pdf]
